# Breath fingerprint of colorectal cancer patients by gas chromatography-mass spectrometry analysis preparatory to e-nose analyses

**DOI:** 10.1016/j.plabm.2025.e00475

**Published:** 2025-05-10

**Authors:** Stefano Dugheri, Ilaria Rapi, Giovanni Cappelli, Niccolò Fanfani, Donato Squillaci, Simone De Sio, Beatrice Mallardi, Paola Mantellini, Fabio Staderini, Veronica Traversini, Antonio Baldassarre, Fabio Cianchi, Nicola Mucci

**Affiliations:** a Department of Life Science, Health, and Health Professions Link Campus University, Rome, Italy; b Department of Experimental and Clinical Medicine, University of Florence, Florence, 50134, Italy; c University of Rome “La Sapienza”, Rome, 00185, Italy; d Oncological Network, Prevention and Research Institute, Florence, 50139, Italy; e Department of Clinical Epidemiology, Cancer Prevention and Research Institute (ISPO), Florence, 50139, Italy; f Department of Surgery and Translational Medicine, University of Florence, 50139, Italy; g Division of Occupational Medicine, Careggi University Hospital, Florence, 50139, Italy

**Keywords:** CRC, Early diagnosis, GC-MS, Electronic nose, Screening test

## Abstract

Colorectal cancer (CRC), according to the most recent data provided by GLOBOCAN, ranks fourth worldwide in incidence and third in mortality among all cancers. Current estimates project a global increase in colorectal cancer incidence of 60.5 % and mortality of 76.9 % between 2022 and 2045. The low sensitivity and adherence, coupled with the high costs associated with current diagnostic methods for CRC, underscore the need to explore innovative procedures for the early detection of tissue abnormalities. Existing research suggests that patients affected by this condition exhibit distinctive alterations in volatile organic compounds (VOCs) ratios in their exhaled breath.

This study presents a characterization of exhaled breath using Gas Chromatography-Mass Spectrometry (GC-MS) in patients with varying stages of the disease, as determined by conventional medical and clinical analyses. An electronic nose was utilized to develop a method aimed at rapidly analyzing a subject's exhaled breath to identify the group of belonging (healthy, affected). The aim of the study was to develop a rapid, cost-effective, and non-invasive early diagnostic system employing an electronic nose. Statistical analysis identified 12 compounds with the potential to distinguish between healthy and affected individuals and were selected for testing the application potential of the Cyranose 320 electronic nose. The ability of the method to identify the 40 subjects analyzed as Healthy Controls (HC) or CRC in terms of sensitivity and specificity (0.8 and 0.85, respectively) demonstrates the feasibility of using this method for rapid, low-cost, and non-invasive disease recognition.

## Highlights

• The analysis of exhaled breath could be a new matrix for CRC screening.
• Breath analysis could increase adherence to current screening methods for CRC.
• GC-MS on exhaled breath allows the identification of potential biomarkers for CRC
• The use of an electronic nose on exhaled breath could distinguish CRC cases.

## Graphical abstract

**Figure:**
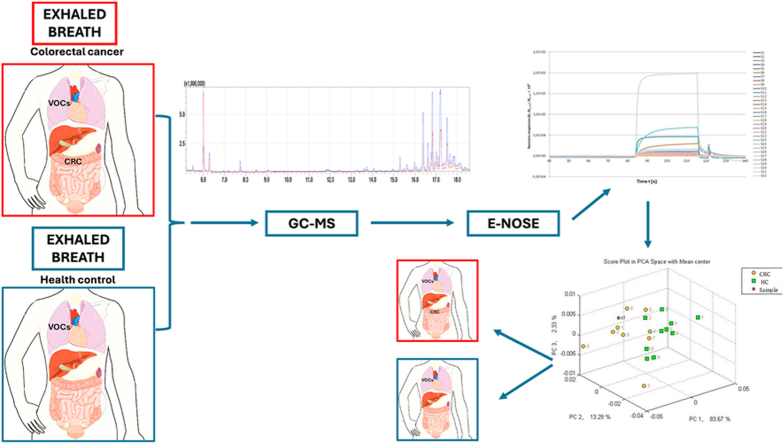

## 1 Introduction

In humans, colorectal cancer (CRC) ranks as the third leading cause of cancer-related mortality and the fourth most common cancer by incidence, with a significant projected increase by 2045 [1,2]. Since the early 2000s, colorectal cancer screening has been included among essential healthcare services, culminating in the “European guidelines for quality assurance in colorectal cancer screening and diagnosis” by the European Commission in 2010 [3]. The primary screening method, recommended and widely used across Europe, is the fecal occult blood test (FOBT) for men and women aged 50 to 74. Additional methods include colonoscopy, flexible sigmoidoscopy, and virtual colonoscopy, each with distinct advantages and limitations. The FOBT, encompassing the guaiac-based test (gFOBT) and the fecal immunochemical test (FIT), is simple and cost-effective. However, it has limited sensitivity and requires colonoscopy for confirmation in positive cases. It has been shown to reduce CRC mortality by 14–16 % [4,5]. Flexible sigmoidoscopy, which can be performed only once in the patient's lifetime, is more accurate for the lower colon and allows immediate polyp removal. However, it has a low adherence rate (21 %) and does not cover the entire colon. Nevertheless, it reduces CRC mortality by 45 % [6]. Colonoscopy is the most comprehensive examination, enabling both diagnosis and treatment. However, it is invasive, costly, and requires complex preparation, making it less acceptable to patients. It is primarily recommended for confirming positive results from other tests or for high-risk individuals [7]. Finally, virtual colonoscopy (CTC) is a non-invasive alternative for selected cases. While it does not allow for biopsies and involves minimal radiation exposure, it is suitable for patients unable or unwilling to undergo traditional colonoscopy [[8], [9], [10]].

Despite the availability of various screening methods and their increasing utilization, participation rates remain low, ranging from 30 % to 70 % depending on the country. Improving adherence is crucial to enhancing patient outcomes [11]. However, these approaches are not always well-received by patients and often involve high costs for national healthcare systems.

Recent research has identified volatile organic compounds (VOCs) in exhaled breath as potential indicators of respiratory and gastrointestinal abnormalities, which may be associated with metabolic changes in tumour cells [[12], [13], [14]]. While VOC presence can also be influenced by external factors such as lifestyle, smoking, or diet, studies have demonstrated a high specificity of VOCs linked to neoplastic tissues [15]. Notably, Ghazal et al. (2021) [16] found that breath analysis is a promising method for early gastric cancer detection, achieving diagnostic sensitivity above 67 % and specificity between 71 % and 98 %. These findings suggest the potential use of VOC analysis as a non-invasive early diagnostic tool for diseases affecting the respiratory and gastrointestinal systems.

Currently, mass spectrometry (MS) remains the most common method for breath analysis and VOC studies [[17], [18], [19], [20], [21]], alongside techniques such as gas chromatography (GC/Q-TOF) [22], selected-ion flow-tube (SIFT-MS) [23], and on-line thermal desorption single-photon ionization (TD-SPI-MS) [24]. More recently, the use of electronic noses has been proposed as an alternative approach for the early detection of respiratory and gastrointestinal cancers. This interest has been bolstered by studies highlighting the ability of trained dogs to detect conditions such as diabetes, COVID-19, and CRC [[25], [26], [27]].

The study of VOCs using electronic noses is a relatively recent development. Electronic noses are instruments equipped with electrochemical sensors that, as an air sample passes through, employ classification algorithms to process the data, enabling qualitative and, in some cases, quantitative evaluation of the analyzed gases. Some devices feature a multi-sensor structure with low selectivity that generates a "smell print", a unique profile of the sample that facilitates its identification. Electronic noses are utilized in various sectors, with food [28] and industrial [29,30] applications being notable examples. They have been applied in the analysis of VOCs associated with processes such as food adulteration, pollution, and air quality control. Subsequently, their use has expanded to medical research, particularly for screening and diagnosing diseases of the upper respiratory tract through exhaled breath analysis [14,31,32]. Their application has further broadened to include other types of cancer, and interest in this field continues to grow in the scientific literature. This growing interest is mirrored economically, with the largest market for portable electronic noses in the United States. The electronic nose market is projected to experience a compound annual growth rate (CAGR) of 10 % from 2023 to 2029, with Europe being the fastest-growing market [33]. As previously mentioned, the applications of electronic noses are highly diverse, spanning military and defence, food and beverage, waste management, environmental monitoring, and healthcare. For the ongoing study, key advantages of these instruments include their high tolerability, rapid analysis capabilities, cost-effectiveness, and strong performance in terms of sensitivity and specificity. The operation of electronic noses generally involves comparing the gas sample to be analyzed with patterns of specific categories, previously identified during the training phase, to classify the newly analyzed sample. The result obtained is a profile given by the response of the different sensors of the instrument, as the exhaled air passes through (identify phase).

This study presents the results of research conducted in polyclinics in central Italy, aimed at the early identification of CRC patients through the analysis of VOCs in exhaled breath. Following a preliminary definition of breath sampling methods for patients with varying degrees of tissue alteration, the study addresses the challenges of characterizing VOCs using conventional GC-MS measurements. It also describes the procedures used to identify the most significant emission peaks for calibrating the electronic nose. The article concludes with an evaluation of the "Smell Print" approach for CRC early diagnosis and discusses its potential applications based on the results obtained from an initial group of patients.

## 2 Methods

The study analyzed the exhaled breath of 40 patients who voluntarily participated, after signing the informed consent, in the research project titled: “Use of Volatile Organic Components (VOC) for the Characterization of Colorectal Diseases (VOC in CRC)” (Ethical Committee approval no. 16770, AV Centro). Participants, aged over 50, were categorized into two groups:

Group A: 20 patients with colorectal cancer awaiting surgical intervention in the clinical surgery department and patients who tested positive for FOBT and confirmed it with colonoscopy.
Group B: 20 individuals who tested negative for FOBT within two years before the analysis and from the gastroenterology department who, following colonoscopy, were healthy.

Subjects over 50 were selected as they are part of the national screening programs due to the highest likelihood of developing CRC.

### 2.1 Sample collection

To prevent contamination of the exhaled breath by ubiquitous environmental VOCs, patients were instructed to inhale for 2 min through a system of filters and adapters, then exhale outside. Following this preparatory phase, three forced exhalations were collected in sealed multi-foil bags. An identification code was used to anonymously analyzed the collected samples. Samples were analyzed using a GC-MS system and a portable electronic nose.

### 2.2 Breath analysis using gas chromatography-mass spectrometry (GC-MS)

The GC-MS analysis was performed using a Shimadzu GCMS-QP2010 system that allows the separation, identification, and quantification of the components of exhaled breath. The combination of GC and MS [34,35]. Through a specific membrane in the bag containing the sample, an SPME (Solid Phase Micro Extraction) fiber Carboxen®/Polydimethylsiloxane (CAR/PDMS 85 μm) is inserted and kept exposed for 10 min. The SPME fiber is a passive sampler that, once inserted into the bag, adsorbs the analytes until equilibrium is reached, depending on the concentration present in the sample [36]. Since no differences were observed, the method was optimized by reducing the analysis times, thus preferring the shortest extraction time.

The adopted instrumental conditions are reported in Table 1.

**Table 1:** GC-MS method configuration.

**Injection port**	Splitless, needle gauge 45 at 290 °C
**Desorption time**	2 min
**Source and Interface temperature**	220 and 260 °C
**Analytical Column**	VF-5ms (Agilent J&W), 5 % phenyl methyl polysiloxane, 60 m × 0.25 mm, ID 1 μm
**Run Time**	36.50 min with start at 3.9 min
**Acquisition**	(SCAN) *m*/*z* 29-300

### 2.3 GC-MS statistical analysis

The GC-MS data obtained for the 40 samples were sorted in the two aforementioned groups and exported to spreadsheet software (Excel) for initial graphical representation and basic statistical analysis using ANOVA, and Statgraphics 19. The area under the curve (AUC) of the chromatographic peaks obtained from the samples was extrapolated in order to evaluate the difference in relative abundance of each peak between the two studied groups. The Wilcoxon Mann-Whitney test was used comparing the data of the two group to identify the statistically significant peaks (p value < 0.05) [37] between an healthy and a affected subject.

The data of GC analysis were additionally elaborated using the Ward method, a multivariate analysis, to evaluate the ability of the chromatographic profile to discriminate between subjects in different groups.

Simultaneously, the identification of the compounds was carried out based on the matching of the mass spectra through the NIST spectral library for the main analytical signals. Previously, a literature research allowed to point out compounds commonly identified as significant markers [19,21,[38], [39], [40], [41]] which are reported in the *Results and Discussion* section. The identification of the compounds was confirmed using commercially available analytical standards: acetone, 2-methylpentane were purchased by Merck KgaA (Darmstad, Germany) and decane 2,2 dimethyl, nonane 1-iodo and octane 4-methyl were purchased by LGC Standards (Manchester, New Hampshire, US).

### 2.4 Calibration of the "CYRANOSE 320″ electronic nose

The electronic nose used was the Cyranose 320, a device manufactured by Sensigent (USA). It consists of 32 nanocomposite array sensors, each featuring two electrodes and a unique insulating polymer containing conductive particles [29,30]. As the gas sample passes over the 32 sensors, it causes swelling and a consequent change in electrical resistance for each of them. The resistance is a function of the type of compound interacting with the sensor, even though the sensors are not specific to individual categories of compounds. The instrument is equipped with the proprietary PCnose and CDAnalysis software, which includes specific functions for converting collected data into a unified response pattern. The tools allow multivariate analysis on five classes, with a maximum of 10 samples per class, where class stands for a homogeneous group of samples with common characteristics.

For this study, a sampling method was developed using two classes with 10 samples per class. The software performs multivariate data analysis using the T^2^ test and generates a summary histogram, illustrating the flow intensity over time during the analysis. This output corresponds to the olfactory impression, enabling a detailed assessment of the sensor array's performance and response dynamics.

### 2.5 Configuration of Cyranose 320 parameters

The Cyranose 320 was configured according to the manufacturer's guidelines, with adjustments tailored to the experimental setup for breath analysis. The main parameters are outlined in Table 2.

**Table 2:** Flow settings and data processing. E-nose.

	Time (s)	Pump Speed
**Baseline Purge**	60	Medium
**Sample Draw2**	40	High
**Snout Removal**	5	/
**1st Air Intake Purge**	60	HIgh
**2nd Sample Gas Purge**	120	High

**Digital Filtering (id/training/calibration)**		on
**Substrate Heater**	**On/Off**	42.0 °C
**Training Repeat Count**		1
**Identifying Repeat Count**		1
**Algorithm**		KNN
**Preprocessing**		Mean-Center
**Normalization**		Normalization 1
**Identification Quality**		Higher
**Acceptance Threshold**		99.90 %

For the developed method, the system was trained by analyzing 20 samples divided into two classes of ten samples each, based on the characteristics of the subjects, in order to create two homogeneous groups: one with a pathological condition of CRC and the other without any alterations (HC). Olfactory signatures were derived and used to classify the analyzed sample. This training phase is crucial for the optimization of the method, as it enhances the instrument's reliability through iterative refinement of its responses.

According to the manufacturer, the classification can be considered reliable if the accuracy exceeds 90 % and the Euclidean distance between classes is greater than five. Upon completing the identification phase, the Cyranose assigns the sample to a class and provides a confidence level for the result, rated on a scale from one to five. The samples used in the training phase of the instrument, given the limited number of individuals available for selection, were chosen based on the pathological condition and supported by results obtained from GC-MS analysis. The chromatographic profile allowed the identification of outliers and facilitated the selection of the samples most representative of the broader population.

## 3 Results and discussion

**Fig. 1:**
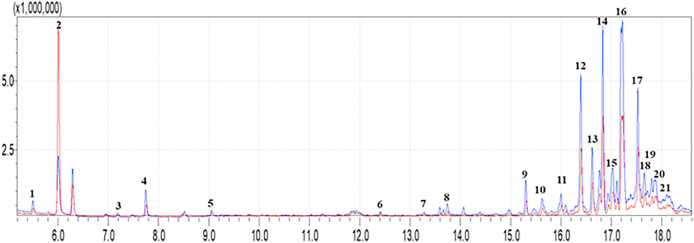
Comparison of the chromatograms from two samples: one HC (in blue) and one with CRC (in red).

The GC-MS analysis revealed significant differences in the mean AUC values of the response associated with the composition of the exhaled air mixture from subjects with or without CRC conditions. Twenty-one peaks were detected with a signal-to-noise ratio above an indicative threshold of three and they were subjected to further evaluation [42]. However, for low molecular weight compounds, it is often not possible to uniquely identify a substance due to the presence of peaks from chemical groups common to substances with similar fragmentation (see Fig. 1).

Twelve peaks (2, 3, 7–9, 11–14 and 16–18) showed statistically significant differences in distinguishing between CRC patients and healthy subjects, with Mann-Whitney test results exceeding 95 % confidence (see Table 3).

**Table 3:** Information on the detected chromatographic peaks and analysis of the differences between CRC and without alteration samples. Significant peaks with p-value less than or equal to 0.05 are shown in bold, the name of the identified samples is shown next to the peak number.

Peak Number/name	RT [min]	*m*/*z*	Healthy controls	CRC	P-value
1	5,5	31,0	4,6⸱10^6^±2,0⸱10^6^	6,7⸱10^6^±6,0⸱10^6^	0,875
**2-acetone**	6,0	43,0	3,6⸱10^6^±9,3⸱10^6^	9,3 × 10^6^±6,5⸱10^6^	0,041
**3**	7,3	43,0	9,2⸱10^5^±6,1⸱10^5^	3,3⸱10^5^±2,5⸱10^5^	0,004
4	7,7	57,0	6,7⸱10^6^±8,4⸱10^6^	4,0⸱10^6^±2,7⸱10^6^	0,468
5	9,0	77,0	5,3⸱10^6^±2,4⸱10^6^	9,5⸱10^6^±1,06⸱10^6^	0,776
6- octane 4-methyl	12,4	43,0	1,2⸱10^6^±8,5⸱10^5^	1,4⸱10^6^±8,6⸱10^5^	0,229
**7**	13,3	43,0	7,8⸱10^5^±1,7⸱10^5^	5,9⸱10^5^±3,6⸱10^5^	0,033
**8**	13,7	57,0	2,3⸱10^6^±1,5⸱10^6^	4,8⸱10^5^±5,0⸱10^5^	0,002
**9**	15,3	57,0	8,0⸱10^6^±3,6⸱10^6^	3,2⸱10^6^±2,0⸱10^6^	0,002
10	15,6	57,0	4,4⸱10^6^±3,8⸱10^6^	2,0⸱10^6^±1,4⸱10^6^	0,073
**11**	16,0	57,0	3,2⸱10^6^±1,4⸱10^6^	1,4⸱10^6^±8,0⸱10^5^	0,002
**12- decane 2,2-dimethyl**	16,4	70,0	4,2⸱10^6^±1,7⸱10^6^	2,0⸱10^6^±1,1⸱10^6^	0,003
**13**	16,6	57,0	2,0⸱10^6^±8,5⸱10^5^	9,8⸱10^6^±5,6⸱10^6^	0,005
**14-nonane 1-iodo**	16,8	57,0	3,4⸱10^6^±1,5⸱10^6^	1,6⸱10^6^±8,5⸱10^6^	0,005
15	17,0	71,0	8,1⸱10^6^±8,5⸱10^6^	3,2⸱10^6^±2,8⸱10^6^	0,054
**16**	17,2	57,0	5,8⸱10^6^±2,5⸱10^6^	3,6⸱10^6^±2,4⸱10^6^	0,029
**17**	17,5	57,0	2⸱10^6^±8,0⸱10^6^	1,1⸱10^6^±5,7⸱10^6^	0,009
**18**	17,65	71,0	4,7⸱10^6^±2,1⸱10^6^	2,7⸱10^6^±1,8⸱10^6^	0,021
19	17,7	73,0	1,1⸱10^6^±4,6⸱10^5^	1,2⸱10^6^±9,2⸱10^5^	>0,05
20	17,9	71,0	6,2⸱10^6^±3,1⸱10^6^	3,5⸱10^6^±2,9⸱10^6^	>0,05
21	18,1	57,0	2,3⸱10^6^±7,8⸱10^5^	1,8⸱10^6^±1,2⸱10^5^	>0,05

The peak 2 corresponding to acetone has a greater expression among subjects with CRC, an element also confirmed in the literature, which points out that it is a secondary product of lipid peroxidation and a predictor for ketosis [43].

Decane 2,2-dimethyl (peak 12) is identified as a key metabolite in the classification of lung cancer (LC) samples in a 2014 study [20]. This study hypothesizes a connection between the production of alkanes, particularly decane 2,2-dimethyl, and processes related to oxidative stress and lipid peroxidation. In 2022, another study proposed a panel of eight VOCs in exhaled breath as potential diagnostic markers for LC, achieving a sensitivity of 0.86 and a specificity of 0.87, with decane 2,2-dimethyl included among the eight compounds [21]. While this compound appears in studies concerning other types of cancer, nonane 1-iodo (peak 14) is cited in scientific articles specifically as a VOC biomarker for CRC, detectable in exhaled breath or fecal samples [38,39]. As most of the studies in literature to date have not identified nonane 1-iodo as a marker for other tumour conditions, its significant specificity for colorectal cancer can be suggested [19]. Finally, octane 4-methyl (peak number 6) is cited as an indicator for the discrimination of CRC patients through GC analysis [40,41] and as a marker in urinary VOC studies for LC [44]. However, there is no agreement regarding its role for CRC since some studies report it as more expressed in one class, while others in the other one. This agrees with what was highlighted in our analysis, where the difference between the HC and CRC groups is not significant.

The rest of the peaks highlighted as significant appear to be associated with alkanes methylated in different positions, such as 2-methylpentane, that are reported in the literature as associated with significant variations between HC and CRC subjects [45].

An intriguing perspective would be to conduct a study aimed at profiling VOCs specific to the exhaled breath of subjects without lesions or comorbidities, comparing them to the exhaled breath of individuals with various types of tumors, such as the four most prevalent ones (breast, prostate, lung, and colorectum). By recruiting a large number of participants, it would be possible to understand the differences in the expression and concentration of these compounds across different types of cancer.

**Fig. 2:**
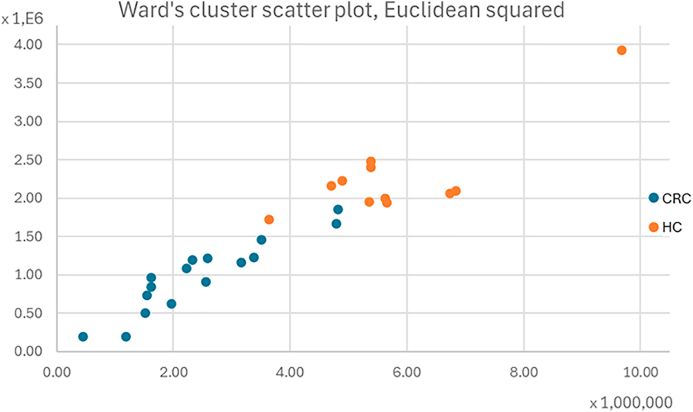
Scatter plot resulting from the multivariate analysis of the subjects with the data relating to the statistically significant peaks (CRC in blue and HC in orange).

The potential of this technique is vast, particularly for exhaled breath analysis. Fig. 2 presents the results of the multivariate analysis and highlights the possibility of clustering the study population into two groups based solely on retention times, AUC, and mass spectra.

In general, the high variability observed in the samples prevents the immediate discrimination of CRC-affected individuals from healthy ones.

It is noteworthy how most of the samples marked in orange corresponding to individuals negative for FOBT, are located on the right side of the graph, while the blue dots, representing individuals with CRC, are clustered on the left. This clearly distinguishes the two populations, which are defined by differing levels of VOC production within the body.

Although GC-MS is an extremely effective tool, it is not optimal for routine screening due to its lengthy analysis times, high costs, and the need for highly trained personnel to operate the instrument and interpret the results. Nevertheless, GC-MS remains the only analytical approach capable of identifying VOCs with high sensitivity and specificity.

**Fig. 3:**
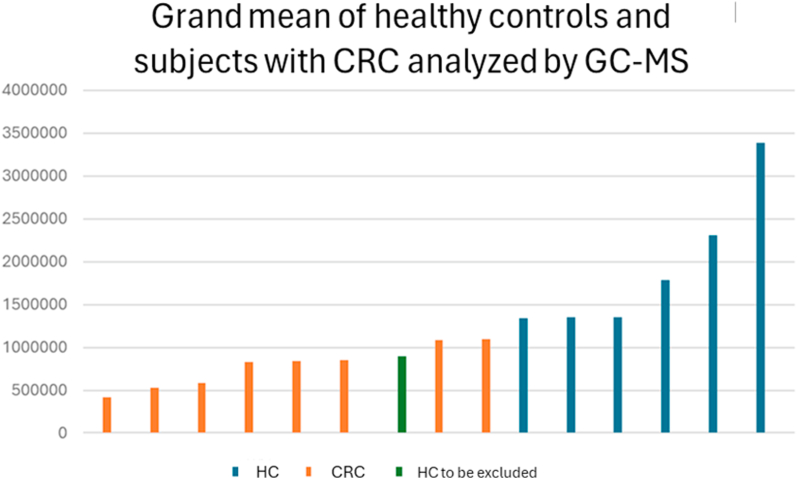
Example of subject distribution through VOC analysis in GC-MS.

**Fig. 4:**
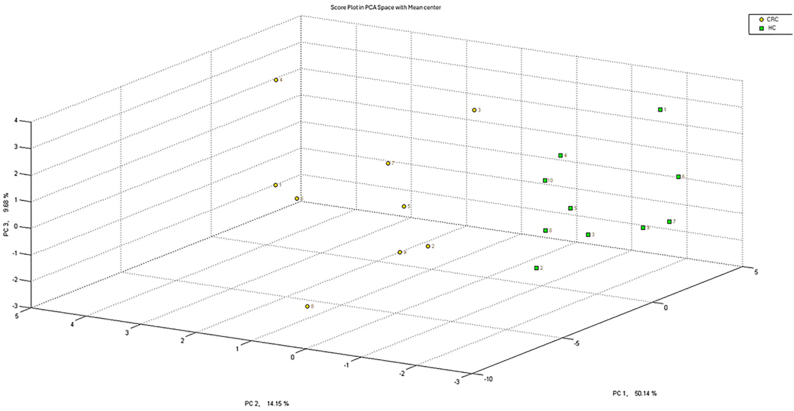
PCA plot of the developed method, spatial distribution of training samples. CRC in yellow, HC in green.

Based on the GC-MS analysis, we were able to improve our understanding of the samples and exclude subjects from the electronic nose training method: only ten samples can be used to create a specific profile in the instrument, thus, the choice of the more representative samples of that class is crucial. [Fig. 3]. By grand the AUC values of the significant peaks, it was possible to identify one HC sample that showed a different profile compared to the rest of the samples in its class (HC). This was likely due to interfering factors, such as inflammatory conditions. This evaluation allowed to exclude it from the training set used to calibrate the electronic nose. Nevertheless, the sample was still included in the analysis alongside the others for the calculation of the method's sensitivity and specificity. Through this, it was possible to develop a method within the electronic nose that included 10 samples from subjects with CRC and 9 from subjects without abnormalities, ensuring compliance with the manufacturer's guidelines. The samples included in the training should therefore be as representative as possible of the most likely conditions found in the population. Including an outlier sample would distort the overall profile of the HCs, compromising the accuracy of the identification part. The internal cross-validation of the method reached 94.7 %, and the Euclidean distance between the classes was greater than 5. The method showed a greater ability to correctly identify the 20 healthy subjects compared to the 20 diseased ones, likely due to the higher variability caused by the pathological condition: the cluster of data of the healthy are well separated from the one of the CRC-subjects [Fig. 4].

**Fig. 5:**
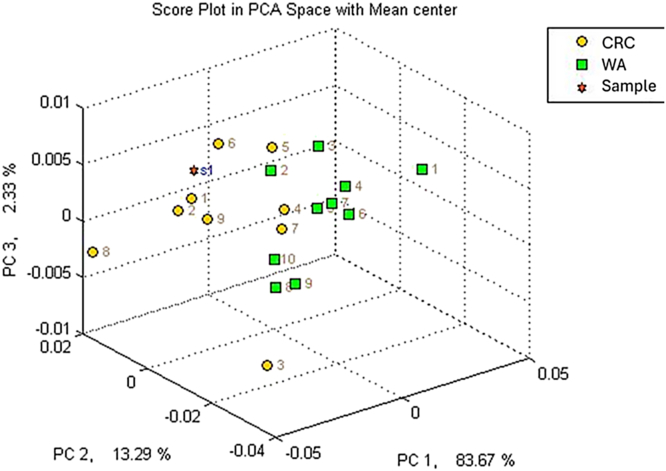
Unknown sample correctly positioned by the system within the CRC class based on the detected profile.

**Fig. 6:**
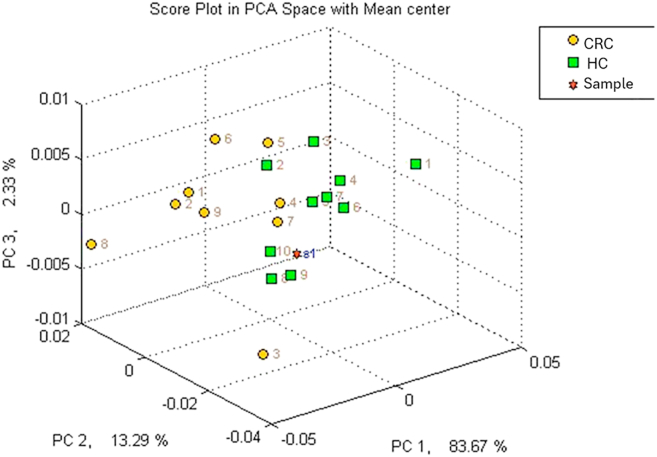
Unknown sample correctly positioned by the system within the HC class based on the detected profile.

To test the method performances, all 40 collected samples were analyzed using the developed method with the electronic nose, showing a sensitivity of 0.8 and a specificity of 0.85. When compared to the current most used screening method, i.e. the FOBT, which shows a sensitivity ranging from 0.54 to 0.83 and a specificity between 0.67 and 0.89 [46], the results appear promising. As shown in Fig. 5, Fig. 6, the sample analysis also generates a graph that highlights a distinct spatial distribution of subjects within their identified class of belonging. The analysis performed with the electronic nose is extremely fast, providing results in less than 5 min. It is also cost-effective and well-tolerated by patients of all ages. Once the optimal method is fully developed, the system will not require specialized personnel for sample collection or operation, presenting itself as an interesting alternative for CRC screening that deserves further exploration.

Although the current sample size is limited, the study displays promising results, and increasing the number of samples will allow for system refinement, leading to improved sensitivity and specificity. Additionally, this will enable a more detailed characterization of subjects and intermediate lesions. Exhaled breath emerges as an interesting, yet largely unexplored, medium with immense potential. Its ease of collection and non-invasive nature make it a matrix that warrants the attention of research centers both nationally and globally, to transition from the realm of research to practical diagnostic applications.

## 4 Conclusions

Given the high incidence and mortality rates of CRC worldwide, along with the low adherence to current screening methods—despite their potential to enable early and often curative interventions—it is evident that further research in this field is urgently needed. The goal is to propose valid alternatives that are well-tolerated by the population. This represents a critical objective to achieve as soon as possible.

The study conducted demonstrated that GC-MS can identify compounds that show significant variability between healthy individuals and patients. Moreover, it allows for the optimization of method development for an innovative system such as the electronic nose. The latter has shown sensitivity and specificity comparable to current CRC screening methods. The preliminary findings suggest that by increasing the number of samples and incorporating additional groups, such as individuals with adenomas, it would be possible to further improve the discriminatory power and include the potential to identify intermediate lesions, such as adenomas.

## CRediT authorship contribution statement

**Stefano Dugheri:** Writing – review & editing, Writing – original draft, Visualization, Supervision, Conceptualization. **Ilaria Rapi:** Writing – review & editing, Writing – original draft, Investigation, Formal analysis, Conceptualization. **Giovanni Cappelli:** Writing – review & editing, Writing – original draft, Investigation, Conceptualization. **Niccolò Fanfani:** Writing – review & editing, Validation, Formal analysis. **Donato Squillaci:** Writing – review & editing, Writing – original draft, Investigation, Data curation. **Simone De Sio:** Writing – review & editing, Data curation. **Beatrice Mallardi:** Writing – review & editing, Validation, Methodology. **Paola Mantellini:** Writing – review & editing, Supervision, Project administration, Data curation. **Fabio Staderini:** Writing – review & editing, Validation, Methodology. **Veronica Traversini:** Writing – review & editing, Visualization, Data curation. **Antonio Baldassarre:** Writing – review & editing, Visualization, Data curation. **Fabio Cianchi:** Writing – review & editing, Supervision, Project administration. **Nicola Mucci:** Writing – review & editing, Supervision, Project administration.

## Ethical statement

The authors are accountable for all aspects of the work in ensuring that questions related to the accuracy or integrity of any part of the work are appropriately investigated and resolved. The study was conducted in accordance with the Declaration of Helsinki (as revised in 2013). The study was approved by the regional ethic board n. 16770 and informed consent was obtained from all individual participants.

## Declaration of competing interest

None.

## Data Availability

The data that has been used is confidential.
